# Solitary fibrous tumor of the prostate with accompanying low-grade prostate cancer

**DOI:** 10.1016/j.eucr.2024.102879

**Published:** 2024-11-06

**Authors:** Marie-Lisa Eich, Kira Furlano, Georg Hilfenhaus, Bernhard Ralla, Ulrich Keilholz, Maria Joosten, Damian T. Rieke, Thorsten Schlomm, David Horst, Simon Schallenberg

**Affiliations:** a Institute of Pathology, Charité Universitätsmedizin Berlin, Corporate Member of Freie Universität Berlin, Humbolt-Universität zu Berlin and Berlin Institute of Health, Berlin, Germany; b Department of Urology Charité - Universitätsmedizin Berlin, Corporate Member of Freie Universität Berlin, Humboldt-Universität zu Berlin and Berlin Institute of Health, Berlin, Germany; c Charité Comprehensive Cancer Center, Charité - Universitätsmedizin Berlin, Corporate Member of Freie Universität Berlin and Humboldt-Universität zu Berlin, Berlin, Germany; d Department of Hematology, Oncology and Cancer Immunology, Campus Charité Mitte, Charité - Universitätsmedizin Berlin, Corporate Member of Freie Universität Berlin and Humboldt-Universität zu Berlin, Berlin, Germany; e Department of Hematology, Oncology and Cancer Immunology, Campus Benjamin Franklin, Charité - Universitätsmedizin Berlin, Corporate Member of Freie Universität Berlin and Humboldt-Universität zu Berlin, Berlin, Germany; f German Cancer Consortium (DKTK), Partner Site Berlin, German Cancer Research Center, Heidelberg, Germany

**Keywords:** Solitary fibrous tumor, SFT, Prostate, Prostate cancer, Multiple primary cancers

## Abstract

We present the rare case of a 51-year-old male diagnosed with a solitary fibrous tumor (SFT) of the prostate, along with a concurrent low-grade prostate adenocarcinoma (Gleason score 3 + 3, Grade Group 1). The diagnosis was confirmed by positive immunohistochemical markers, including CD34, BCL2, and STAT6, and molecular analysis showing a NAB2-STAT6 fusion. Following successful surgical management and the simultaneous diagnosis of a pulmonary relapse from a prior thyroid carcinoma, the patient remains under clinical surveillance. This is particularly significant given the patient's history of multiple tumors, including Hodgkin's lymphoma, papillary thyroid carcinoma, prostate cancer, and SFT.

## 1 Introduction

Solitary fibrous tumors (SFTs) are rare mesenchymal neoplasms first described in the pleura.^1^ They are most frequently detected at the extremities, in deep soft tissue and pleura, but can occur at any anatomical site.^2^^,^^3^ SFTs of the prostate are uncommon and are often discovered incidentally.^4^ These tumors can be challenging to diagnose on biopsy due to overlapping histological features with other spindle-cell neoplasms. It is hypothesized that these tumors originate from fibroblastic mesenchymal stem cells^5^ and show characteristic *NAB2*-*STAT6* fusions, alongside haphazardly arranged spindle cells and thin-walled staghorn vessels.^2^

## 2 Case presentation

**Fig. 1:**
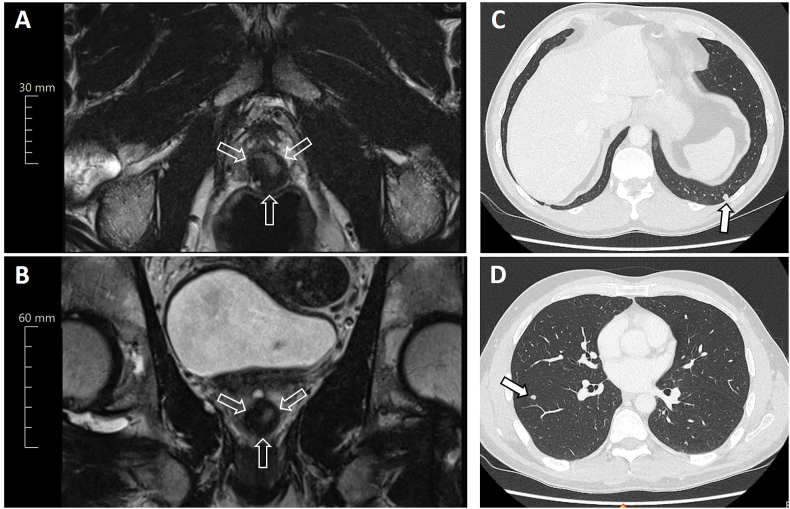
Pelvic magnetic resonance imaging shows a 2 cm prostatic mass on (A) axial and (B) sagittal T2-weighted images as indicated by open arrows. (C and D) Subsequent chest CT imaging reveals multiple suspicious bipulmonary nodules (maximum diameter 8 mm; indicated by filled arrows).

A 51-year-old male with a past medical history of Hodgkin's lymphoma (diagnosed in 1987), papillary thyroid carcinoma (diagnosed and resected in 1995), and basal cell carcinoma of the skin (2015), presented for routine prostate cancer screening. The patient's prostate-specific antigen (PSA) level was 1.13 ng/ml, which is within the normal range. However, transrectal digital examination followed by a transrectal ultrasound showed suspicious findings, necessitating further investigation. A multiparametric magnetic resonance imaging (mpMRI) scan demonstrated a 2 cm T2 hypointense mass, located dorsomedian in the peripheral zone, extending from the apex to the midgland (see Fig. 1). The mass was not considered highly suspicious for prostate cancer and was classified as PI-RADS 3.

**Fig. 2:**
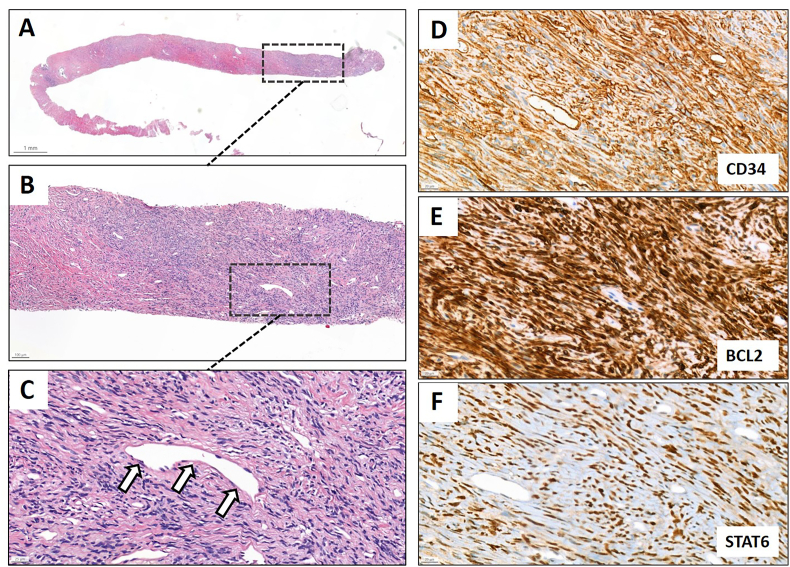
Prostatic biopsy showing (A) low-power and (B) intermediate-power H&E staining of a haphazardly arranged spindle cell lesion with (C) staghorn vessels (open arrows). The lesion is (D) positive for CD34, (E) BCL2 and (F) STAT6 on immunohistochemical staining.

A perineal MRI fusion prostate biopsy was conducted at an external medical facility. Hematoxylin and eosin (H&E) staining revealed a dense, haphazardly arranged spindle cell lesion with mild nuclear atypia (see Fig. 2A and B) and staghorn vessels (see Fig. 2C). Immunohistochemical staining confirmed the SFT diagnosis, with positive results for CD34, BCL2, and STAT6 (see Fig. 2D–F), the latter showing strong and diffuse nuclear staining. The lesion was negative for desmin and smooth muscle actin (SMA). As part of our specialized prostate and sarcoma tumor board conference, the patient was referred to our hospital for a second medical opinion. Subsequently, the tissue specimen was sent to our pathology department for a thorough histopathological review, providing an accurate second assessment.

As part of the preoperative assessment, a staging CT scan of the chest revealed multiple suspicious bipulmonary nodules with a maximum diameter of 8 mm (see Fig. 1C and D). To differentiate between primary lung tumors and distant metastasis of the prostate SFT, a video-assisted thoracic surgery was performed. Two small nodules of the right lower lobe were resected. Surprisingly, histopathological workup revealed a late pulmonary relapse of papillary thyroid carcinoma by positive expression of thyreoglobulin, CK7, and nuclear TTF-1. Given the good prognosis of the metastatic thyroid carcinoma indicated by a low Ki-67 proliferation index of 1 % and an effective treatment modality by radioactive iodine therapy, our prostate tumor board reaffirmed their initial recommendation of surgical resection of the localized SFT by prostatectomy. Consequently, a follow-up MRI of the prostate was conducted showing a slight growth of the known SFT lesion, with an increase of less than 20 % over the one year period. Despite this minor increase in size, the mass's signal behavior remained unchanged.

Treatment options were carefully evaluated by the interdisciplinary tumor board. The gold standard treatment for SFT is surgical resection. Active surveillance and focal therapy were discussed as alternative approaches. However, due to the tumor's location within the prostate and the potential risks of recurrence or malignant transformation, the multidisciplinary team, in consultation with the patient, decided on surgical resection with negative margins to minimize recurrence risk and ensure complete removal.

**Fig. 3:**
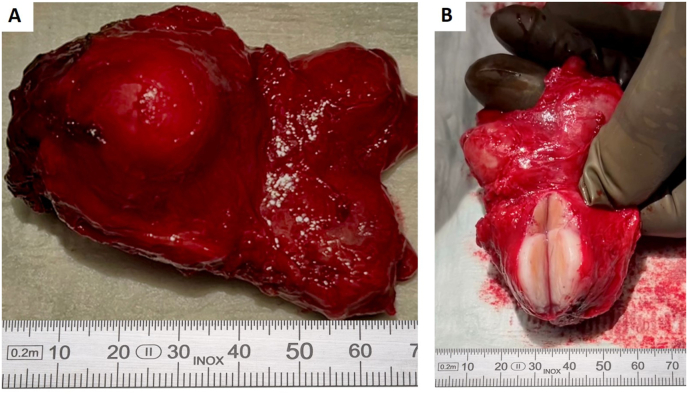
Gross specimen of prostatectomy with solitary fibrous tumor. (A) Prostatectomy specimen with a prominent solitary fibrous tumor. The tumor is well-circumscribed, displaying a smooth, bulging surface. Adjacent prostate tissue is evident. (B) The specimen is sectioned, revealing the internal architecture of the solitary fibrous tumor. The cut surface demonstrates a firm, fibrous texture, characteristic of solitary fibrous tumors, with clear demarcation from the surrounding prostate tissue. A measuring scale (in mm) is included for size reference in both panels.

**Fig. 4:**
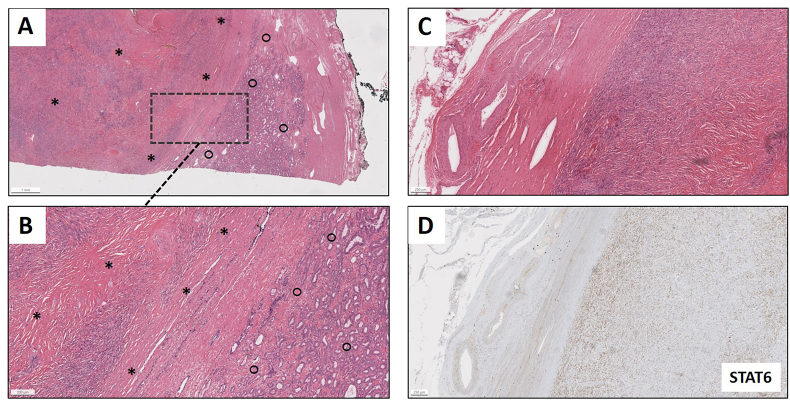
Prostatectomy specimen showing (A) low-power and (B) intermediate power H&E staining of a spindle cell lesion (solitary fibrous tumor; area indicated by asterisks) adjacent to an acinar adenocarcinoma of the prostate (area indicated by circles). (C) H&E staining of the spindle cell lesion, which (D) exhibits a nuclear expression of STAT6 by immunohistochemical staining.

Given the patient's age and the tumor's proximity to neurovascular bundles, nerve-sparing was carefully considered. However, a preoperative digital rectal examination (DRE) revealed a suspicious finding, which was confirmed intraoperatively by the surgeon, who noted a palpable abnormality extending laterally. Consequently, the surgical team aimed for an open en bloc resection without nerve-sparing to prioritize complete tumor clearance (see Fig. 3A and B). The surgical specimen contained a 23-mm spindle cell lesion, which was completely excised (see Fig. 4A–C). Immunohistochemical straining for STAT6 (see Fig. 4D) and fusion analysis using the ArcherDX FusionPlex sarcoma kit, showing a *NAB2* (exon 4)-*STAT6* (exon 2) fusion, corroborated the biopsy diagnosis of SFT. The SFT showed a low mitotic rate (1/mm^2^) and a Ki-67 proliferation index of 1–2%, indicative of a low proliferative activity. The tumor was classified as low-risk (age <55, 1 mitosis/mm^2^, <5 cm in size, no necrosis; score 1) according to both the 3-variable and 4-variable classifications for predicting metastatic risk in SFTs.^2^^,^^3^^,^^6^

Unexpectedly, the final pathology report indicated an accompanying acinar prostatic adenocarcinoma adjacent to the SFT (see Fig. 4A and B), with a stage of pT2b, no lymph node involvement (pN0 (0/9)) and no lymphovascular or perineural invasion (L0 V0 Pn0), with a Gleason score 3 + 3 = 6 (Grade Group 1), consistent with a low-risk profile. Molecular analysis using targeted panel sequencing of 624 genes, the tumor showed a pathogenic *LRP1B* (c.9626-2A > T) mutation with an allele frequency of 5.61 %.

The patient's postoperative recovery was uneventful and radioactive iodine therapy of the additional metastatic thyroid cancer was performed in the following weeks.

At the most recent follow-up, one year postoperatively, the patient remained asymptomatic, with no evidence of SFT or prostate cancer recurrence based on imaging. The patient's postoperative PSA levels have remained stable and undetectable, with the most recent measurement in September 2024 at <0.01 ng/ml.

Given the gradual diagnosis of 5 distinct tumor types for this individual (Hodgkin's lymphoma, thyroid carcinoma, basal cell carcinoma, SFT and prostatic adenocarcinoma), we referred the patient to our human genetics department. However, beside the detection of a variant of uncertain significance (VUS) in the MSH6 gene (NM_000179.3:c.3848T > C), a known tumor predisposition syndrome-associated germline mutation could not be detected using a sequencing panel of 28 most relevant genes.

## 3 Discussion

Solitary fibrous tumors of the prostate are exceedingly rare, with few case reports and a small case series documented in the literature.^4^^,^^7^, ^8^, ^9^ The key immunohistochemical stains for the diagnosis of SFT are CD34, BCL2, and STAT6.^2^ STAT6 nuclear staining is particularly crucial as it indicates the *NAB2*-*STAT6* gene fusion, which is characteristic of SFTs.^10^ Additionally, in our case, we confirmed the diagnosis by fusion analysis. Diagnostic challenges arise because SFTs can resemble other spindle-cell lesions, including prostatic stromal sarcoma, stromal tumor of uncertain malignant potential, sarcomatoid carcinoma, and gastrointestinal stromal tumors (GIST).^11^

Prostatic SFTs generally follow an indolent course with a few cases of malignant SFT within the prostate being reported.^7^^,^^12^^,^^13^ To date, only one patient with a prostatic SFT has experienced recurrence, which was likely due to an initial incomplete resection of the tumor^14^^,^^15^. Additionally, there is one reported case of metastatic disease in an obturator lymph node^12^. Therefore, complete surgical excision with negative margins is the main treatment strategy to minimize the risk of recurrence.

The patient, who underwent radical prostatectomy, remains recurrence-free at one-year follow-up; however, erectile dysfunction has been observed due to en bloc resection without nerve sparing. Prognostically, factors such as mitotic rate, tumor size, and necrosis are of significant importance.^2^^,^^6^ Our patient's tumor, with a low mitotic rate, no necrosis, and size of 23 mm, indicates a low risk for recurrence and metastasis. The low Ki-67 proliferation index provides further evidence to support the indolent nature of the tumor.

In the prostate resection specimen, a low-grade prostate adenocarcinoma was identified in direct proximity to the SFT. Thus, our case represents a rare incident of two distinct primary tumor types side by side in the same primary organ. As collision tumors are extremely rare, most studies only describe the clinical and pathological parameters and do not provide any functional insides^16^. How these two unique and adjacent tumor microenvironments in our case mutually affect the distinct biology of each tumor by direct cell-cell interactions and local cytokine gradients, potentially promoting invasiveness and distant metastasis, remains to be explored.

Overall, our case underscores the importance of a rigorous histopathological evaluation using biopsy and/or surgical resection of suspicious lesions in cancer patients, as the incidence of multiple primary tumors is reported in the literature in the range of 2–17 %.^17^ For our patient this approach reconfirmed the SFT diagnosis, and in addition revealed the diagnosis of a localized prostate adenocarcinoma and a pulmonary relapse of a former thyroid carcinoma. Considering the patient's past medical history with a total of 5 different tumor types, the possibility of a hereditary cause was raised. However, a causative germline mutation was not detected in this case. Nevertheless, there is an elevated risk of developing secondary malignancies following treatment for Hodgkin's lymphoma. Hence, the incidence of thyroid carcinoma and soft tissue sarcomas is increased in this patient population, while this does not apply to the risk of prostate cancer development.^18^

## 4 Conclusion

This case is an exceedingly rare report of a solitary fibrous tumor of the prostate coexisting with a low-grade prostate adenocarcinoma. It illustrates the utility of immunohistochemical markers, particularly STAT6, and fusion analysis in diagnosing rare spindle-cell tumors in the genitourinary tract. Furthermore, it highlights the importance of a rigorous histopathological assessment by resection of suspicious lesions due to the possibility of multiple primary tumors. The patient's successful outcome following complete resection underscores the importance of early intervention and highlights the generally indolent nature of both pathologies in this case. Continued follow-up is essential to monitor for any potential recurrence, especially given the patient's past medical history of Hodgkin's lymphoma and papillary thyroid carcinoma.

## CRediT authorship contribution statement

**Marie-Lisa Eich:** Writing – review & editing, Writing – original draft, Visualization, Data curation. **Kira Furlano:** Writing – review & editing, Writing – original draft, Visualization, Data curation. **Georg Hilfenhaus:** Writing – review & editing, Writing – original draft, Visualization, Data curation. **Bernhard Ralla:** Writing – review & editing, Supervision. **Ulrich Keilholz:** Writing – review & editing, Resources, Conceptualization. **Maria Joosten:** Writing – review & editing, Formal analysis. **Damian T. Rieke:** Writing – review & editing, Supervision. **Thorsten Schlomm:** Writing – review & editing, Supervision, Resources. **David Horst:** Writing – review & editing, Resources. **Simon Schallenberg:** Writing – review & editing, Supervision, Conceptualization.

## Declaration of competing interest

The authors declare that they have no known competing financial interests or personal relationships that could have appeared to influence the work reported in this paper{Citation}
